# Clinical Efficacy and Safety of Nivolumab in Japanese Patients With Malignant Pleural Mesothelioma: 3-Year Results of the MERIT Study

**DOI:** 10.1016/j.jtocrr.2020.100135

**Published:** 2020-12-29

**Authors:** Nobukazu Fujimoto, Morihito Okada, Takashi Kijima, Keisuke Aoe, Terufumi Kato, Kazuhiko Nakagawa, Yuichiro Takeda, Toyoaki Hida, Kuninobu Kanai, Jun Hirano, Yuichiro Ohe

**Affiliations:** a Department of Medical Oncology, Okayama Rosai Hospital, Okayama, Japan; b Department of Surgical Oncology, Research Institute for Radiation Biology and Medicine, Graduate School of Biomedical and Health Sciences, Hiroshima University, Hiroshima, Japan; c Division of Respiratory Medicine, Hyogo College of Medicine, Nishinomiya, Japan; d Department of Medical Oncology and Clinical Research, Yamaguchi-Ube Medical Center, Ube, Japan; e Department of Thoracic Oncology, Kanagawa Cancer Center, Yokohama, Japan; f Department of Medical Oncology, Kindai University Faculty of Medicine, Osakasayama, Japan; g Department of Respiratory Medicine, National Center for Global Health and Medicine, Tokyo, Japan; h Department of Thoracic Oncology, Aichi Cancer Center Hospital, Nagoya, Japan; i Department of Pulmonary Medicine and Oncology, Wakayama Medical University, Wakayama, Japan; j Oncology Clinical Development Planning I, Oncology Clinical Development Unit, Ono Pharmaceutical Co., Ltd., Osaka, Japan; k Department of Thoracic Oncology, National Cancer Center Hospital, Tokyo, Japan

**Keywords:** Malignant pleural mesothelioma, Nivolumab, Programmed death-1, Japan

## Abstract

**Introduction:**

We examined the long-term efficacy and safety of nivolumab, a human monoclonal antibody that inhibits interactions between the programmed cell death protein-1 receptor and its ligands (programmed death-ligand 1 and programmed death-ligand 2), in Japanese patients with malignant pleural mesothelioma (MPM).

**Methods:**

Japanese patients with previously treated MPM (one or two regimens) were enrolled in a single-arm, phase 2 study and received nivolumab intravenously 240 mg every 2 weeks until progressive disease or unacceptable toxicity. The primary end point was the centrally assessed objective response rate. Other end points included overall survival (OS), progression-free survival (PFS), treatment-related adverse events, and patient-reported outcomes (Lung Cancer Symptom Scale for mesothelioma and EuroQOL visual analog scale). Patient enrollment started on June 16, 2016. Here, we report 3-year follow-up data (cutoff date: November 12, 2019).

**Results:**

Thirty-four patients were enrolled. The centrally assessed objective response rate was previously reported (29.4%). The 2- and 3-year OS rates were 35.3% and 23.5%, respectively, and the corresponding PFS rates were 17.0% and 12.7%. Median OS and PFS were 17.3 and 5.9 months, respectively. Eight patients were alive at 3 years of follow-up. Nivolumab was well tolerated and no new safety signals were found. The patient-reported outcomes were maintained without marked deteriorations during the study.

**Conclusions:**

Our results reveal clinically relevant long-term efficacy and safety of nivolumab for the treatment of MPM.

## Introduction

Malignant pleural mesothelioma (MPM) is a rare, highly aggressive malignancy that is mostly due to occupational exposure to asbestos and is more common in older males.^1^, ^2^, ^3^ In previous Japanese studies, the median survival of patients with newly diagnosed MPM was just 7.9 months, generally because most patients are diagnosed at an advanced stage.^1^^,^^2^ The U.S. National Comprehensive Cancer Network guidelines for MPM recommend pemetrexed plus cisplatin (or carboplatin) with or without bevacizumab as first-line chemotherapy.^4^ However, most patients fail to respond to first-line chemotherapy, necessitating subsequent systemic therapy, which may now involve pemetrexed (if not administered as first-line chemotherapy or as rechallenge), nivolumab with or without ipilimumab, or pembrolizumab.^4^

Nivolumab, a human monoclonal antibody that inhibits interactions between the programmed cell death protein-1 receptor and its ligands (programmed death-ligand 1 [PD-L1] and PD-L2), was approved in Japan (August 2018) for patients with pemetrexed–platinum doublet-treated MPM on the basis of the results of the Multicenter, Open-label, Single-arm, Japanese Phase II study in Malignant Pleural Mesothelioma (MERIT) study,^5^ which enrolled 34 Japanese patients. After a median follow-up of 16.8 months, 10 patients had an objective response and the median overall survival (OS) was 17.3 months.^5^

To our knowledge, there are no published studies reporting the 3-year OS after second-line treatment. Here, we report the results obtained at the 3-year follow-up of patients enrolled in the MERIT study, including the efficacy outcomes for all patients and according to PD-L1 expression and MPM subtype (epithelioid or nonepithelioid), changes in quality of life (QOL) (determined using the EuroQOL visual analog scale [EQ-VAS] and Lung Cancer Symptom Scale for mesothelioma [LCSS-Meso] average symptom burden index), and treatment-related adverse events (TRAEs).

## Materials and Methods

MERIT was an open-label, single-arm, phase 2 study performed at 15 centers in Japan. Its design is described in more detail in our previous report.^5^ This study adhered to the Declaration of Helsinki and Good Clinical Practice and was registered on clinicaltrials.jp (JapicCTI-163247).

### Patients

The full eligibility criteria are described in our previous report.^5^ Briefly, males and females aged at least 20 years were eligible if they had histologically confirmed MPM, unresectable advanced or metastatic MPM without surgery, MPM resistant or intolerable to one or two previous chemotherapeutic regimens (platinum and pemetrexed), and at least one measurable lesion defined according to the modified Response Evaluation Criteria in Solid Tumors (mRECIST) for MPM.^6^ Key exclusion criteria included history of severe hypersensitivity reactions to other drugs (including antibody products), concurrent or history of autoimmune disease, multiple primary cancers, brain or meningeal metastases, current or history of interstitial lung disease or pulmonary fibrosis, and previous treatment with immune checkpoint inhibitors (ICIs), therapeutic antibodies, or drugs targeting T-cell regulation. All patients provided written informed consent for participation in the study.

### Study Design

All patients were treated with nivolumab at a dose of 240 mg by intravenous infusion every 2 weeks (one cycle) on day 1 of each cycle. Its dose or administration mode could not be adjusted. As previously explained,^5^ nivolumab was to be continued until the patient met one of the discontinuation criteria: documentation of progressive disease (PD); unequivocal clinical progression; grade 2 or higher interstitial lung disease, grade 2 or higher eye disorder that did not improve to grade 1 or less with topical treatment, and a causal relationship with nivolumab could not be excluded; grade 3 or higher bronchospasm, neurotoxicity, hypersensitivity reaction, infusion reaction, or uveitis for which a causal relationship with nivolumab could not be excluded; no administration of nivolumab for 6 weeks after the previous dose (unless nivolumab is withheld for at least 6 weeks for steroid tapering); or the investigator or subinvestigator deemed it necessary to discontinue treatment in consideration of the efficacy or safety of nivolumab. Immunosuppressants, corticosteroids at doses of at least 10 mg/day prednisone equivalents, antitumor therapies, concurrent radiotherapy, pleurodesis, and surgical therapies for malignant tumors were prohibited. Tumor imaging (computed tomography or magnetic resonance imaging) was performed every three cycles. Target lesions in the pleura were measured unidimensionally as the largest tumor thickness perpendicular to the chest wall or mediastinum according to mRECIST.^6^ Nonpleural lesions were measured according to RECIST version 1.1. PD-L1 expression was assessed as previously described.^5^ PD-L1–positive status was defined as membranous staining in at least 1% of tumor cells.

### End Points

The primary end point was the objective response rate (ORR), with central assessment according to mRECIST, and was defined as the proportion of patients with a complete response or partial response (PR). Secondary end points included the investigator-assessed ORR, changes in tumor size, disease control rate, OS, progression-free survival (PFS), duration of response, time to response, and best overall response (BOR) assessed centrally. Tumor responses were assessed in all patients combined and in patients divided into subgroups by PD-L1 expression (<1% or ≥1%) and histologic subtype (epithelioid, sarcomatoid, or biphasic) in prespecified analyses. QOL was assessed using the EQ-VAS and the LCSS-Meso symptom burden index^7^ at enrollment and at each study visit. Safety was evaluated in terms of laboratory tests, AEs, and TRAEs. AEs and TRAEs were graded according to the National Cancer Institute Common Terminology Criteria for Adverse Events, version 4.0.

### Statistical Analyses

As previously noted, a sample size of at least 29 patients was sufficient to detect a significant ORR with a power of 80% and a one-sided significance level of 0.025, on the basis of an expected ORR of 19%.^5^ We also performed a landmark analysis of OS according to the BOR at 3 months for patients who survived for at least 3 months. All analyses were performed using standard methods at 95% confidence levels. Wilson’s method was used to determine the 95% confidence intervals (CIs) for the ORR, disease control rate, and BOR. All analyses were conducted using SAS version 9.3 (SAS Institute Inc., Cary, NC).

### Role of the Funding Source

This work was funded by Ono Pharmaceutical Co., Ltd., Japan, and Bristol-Myers Squibb, United States. The sponsors contributed to the study design, data collection, data analysis, data interpretation, and writing of the clinical study report.

## Results

### Patients

Patient enrollment started on June 16, 2016, and patients were followed up to the data cutoff date, November 12, 2019. Forty-three patients were screened (provided consent), and nine were excluded because they did not meet the inclusion criteria or withdrew their consent. A total of 34 patients were enrolled and treated with nivolumab, including 29 males (85.3%) and five (14.7%) females. Their characteristics are described in Supplementary Table 1 and in our previous report.^5^ The minimum follow-up was 36 months. The median follow-up was 17.3 (range: 1.8–39.9) months for all 34 patients and 38.0 (range: 37.0–39.9) months for seven censored patients included in the end-of-study analysis.

### Overall Response Rate

The centrally assessed ORR was unchanged from our previous report at 29.4% (95% CI: 16.8%–46.2%; 10 of 34 patients), with PR in 10 patients (Table 1). In most patients with PR or stable disease, their responses were maintained for a long period of time (Supplementary Fig. 1), up to approximately 2 years. Table 1 reveals the ORR in subgroups of patients, including the previously reported ORR by histologic subtype and PD-L1 status.^5^ The present analyses newly revealed that the ORR was lower in patients with two previous treatments than in patients with one previous treatment.

**Table 1:** Responses to Nivolumab (N = 34)

Outcome	n/N (%)^a^	95% CI
BOR		
CR	0/34 (0.0)	0.0–10.2
PR	10/34 (29.4)	16.8–46.2
Stable disease	13/34 (38.2)	23.9–55.0
PD	9/34 (26.5)	n/c
NA	2/34 (5.9)	n/c
Response rate by subgroup		
Sex		
Male	7/29 (24.1)	12.2–42.1
Female	3/5 (60.0)	23.1–88.2
Age (y)		
<65	3/11 (27.3)	9.7–56.6
≥65	7/23 (30.4)	15.6–50.9
ECOG PS		
0	4/13 (30.8)	12.7–57.6
1	6/21 (28.6)	13.8–50.0
Histologic subtype		
Epithelioid	7/27 (25.9)	13.2–44.7
Sarcomatoid	2/3 (66.7)	20.8–93.9
Biphasic	1/4 (25.0)	4.6–69.9
Number of prior treatment(s)		
1	9/24 (37.5)	21.2–57.3
2	1/10 (10.0)	1.8–40.4
PD-L1 status		
≥1%	8/20 (40.0)	21.9–61.3
<1%	1/12 (8.3)	1.5–35.4
NA	1/2 (50.0)	9.5–90.5

BOR, best overall response; CR, complete response; ECOG PS, Eastern Cooperative Oncology Group performance status; NA, not assessable; n/c, not calculable; PD, progressive disease; PD-L1, programmed death ligand-1; PR, partial response.

a Percentages are calculated by the number (N) of patients within that subgroup.

### OS and PFS

**Figure 1:**
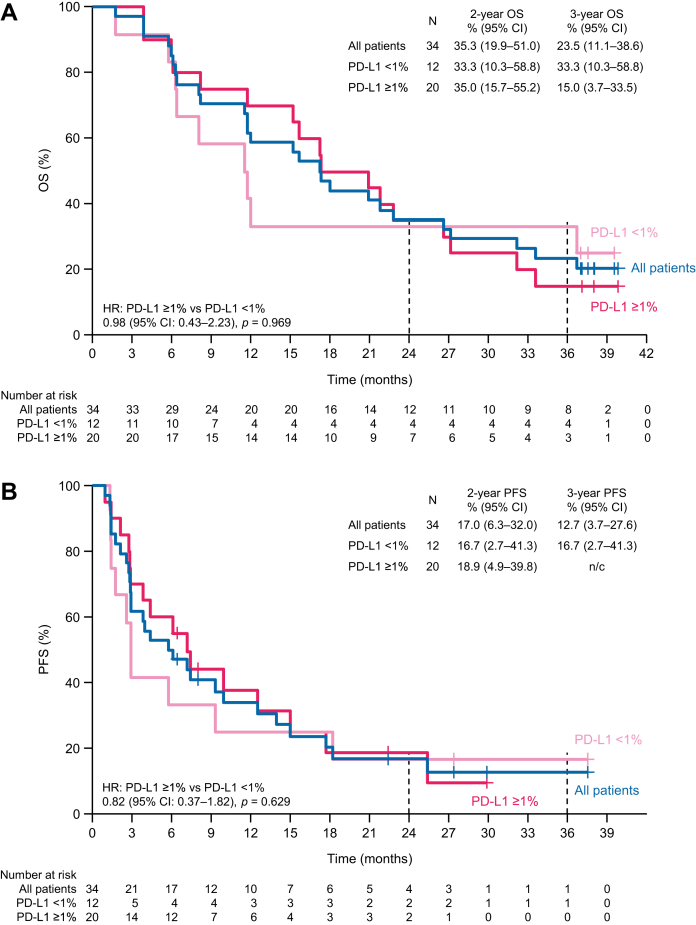
(*A*) OS and (*B*) PFS in all patients and in patients divided into subgroups by PD-L1 expression. CI, confidence interval; HR, hazard ratio; n/c, not calculable; OS, overall survival; PD-L1, programmed death-ligand 1; PFS, progression-free survival.

The 2- and 3-year OS rates were 35.3% and 23.5%, respectively, and the median OS was 17.3 months (95% CI: 11.5–26.6 months) (Fig. 1*A*). The 2- and 3-year PFS rates were 17.0% and 12.7%, respectively, and the median PFS was 5.9 months (Fig. 1*B*).

In PD-L1–positive patients, the 2- and 3-year OS rates were 35.0% and 15.0%, respectively, and the median OS was 19.1 months. The 2- and 3-year PFS rates were 18.9% and not calculable, respectively, and the median PFS was 7.2 months. In PD-L1–negative patients, the 2- and 3-year OS rates were both 33.3%, and the median OS was 11.6 months. The 2- and 3-year PFS rates were both 16.7%, and the median PFS in this subgroup was 2.9 months.

**Figure 2:**
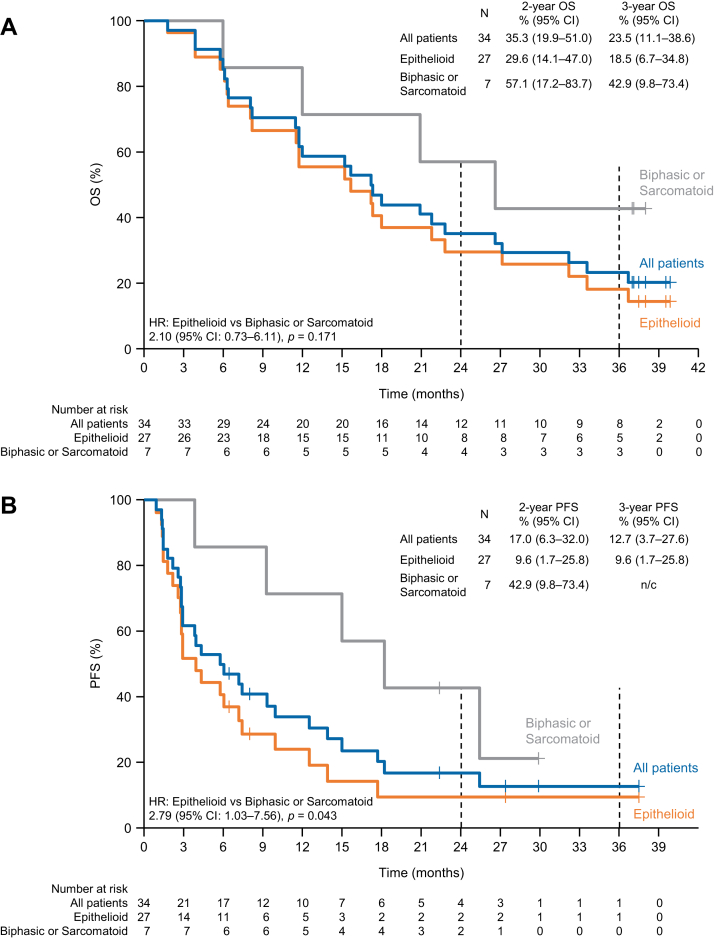
(*A*) OS and (*B*) PFS according to histologic subtype. Patients with biphasic or sarcomatoid histologic subtypes were pooled and compared with patients with the epithelioid histologic subtype. CI, confidence interval; HR, hazard ratio; n/c, not calculable; OS, overall survival; PFS, progression-free survival.

OS and PFS according to the histologic subtype of MPM are shown in Figure 2. Owing to the small numbers of patients with sarcomatoid or biphasic histologic subtypes, these patients were pooled together (as nonepithelioid subtype). In this subgroup, the median OS was 26.6 months, with 2- and 3-year OS rates of 57.1% and 42.9%, respectively (Fig. 2*A*). The median PFS was 18.2 months, whereas 2- and 3-year PFS rates were 42.9% and not calculable, respectively (Fig. 2*B*). In patients with the epithelioid histologic subtype, the median OS was 15.7 months and the 2- and 3-year OS rates were 29.6% and 18.5%, respectively (Fig. 2*A*). The median PFS, 2-year PFS, and 3-year PFS were 3.9 months, 9.6%, and 9.6%, respectively (Fig. 2*B*).

We also performed a landmark analysis of OS in patients with a best response of PR, stable disease, or PD (Supplementary Fig. 2). The median OS in these three subgroups was 20.9, 19.9, and 8.0 months, respectively.

### Patient Status at 3 Years and Poststudy Treatments

**Figure 3:**
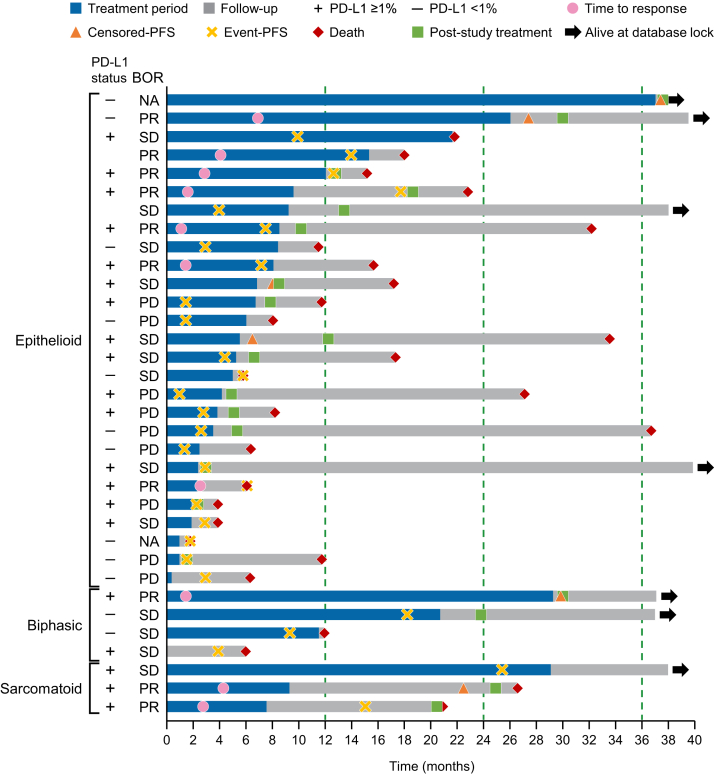
Swimmer plot of treatment duration, response to nivolumab, and follow-up period. BOR, best overall response; NA, not assessable; PD, progressive disease; PD-L1, programmed death-ligand 1; PFS, progression-free survival; PR, partial response; SD, stable disease.

Eight patients were alive at 3 years of follow-up, including seven at the database lock (Fig. 3). These seven patients were on a poststudy treatment at the cutoff date. They included four with epithelioid, two with biphasic, and one with sarcomatoid histologic subtypes. Four patients were treated with nivolumab for 2 years and one patient for 3 years. Eighteen patients received subsequent systemic treatments, as listed in Supplementary Table 2, including nivolumab in three patients. Nivolumab was not rechallenged as subsequent treatment in patients with PD, but one patient was switched to commercially available nivolumab after completing the clinical study, one patient started on commercially available nivolumab after the patient requested discontinuation of the clinical study upon approval of nivolumab in Japan, and one resumed nivolumab after discontinuation due to an AE.

### Comparison of 3-Year Survivors and Nonsurvivors

In an exploratory analysis, we compared the characteristics and BOR between patients who survived for 3 years and nonsurvivors (Supplementary Table 3). Although there was an imbalance in the numbers of patients in these two groups, we observed no marked differences in their patient characteristics, except for the distribution of Eastern Cooperative Oncology Group performance status (ECOG PS) of 0 and 1, with a significantly higher proportion of patients with ECOG PS of 0 among 3-year survivors (*p* = 0.033). The proportion of patients with a BOR of PR or stable disease was not significantly different between the two groups (75.0% in 3-year survivors and 65.4% in nonsurvivors, *p* = 0.640).

### Quality of Life

**Figure 4:**
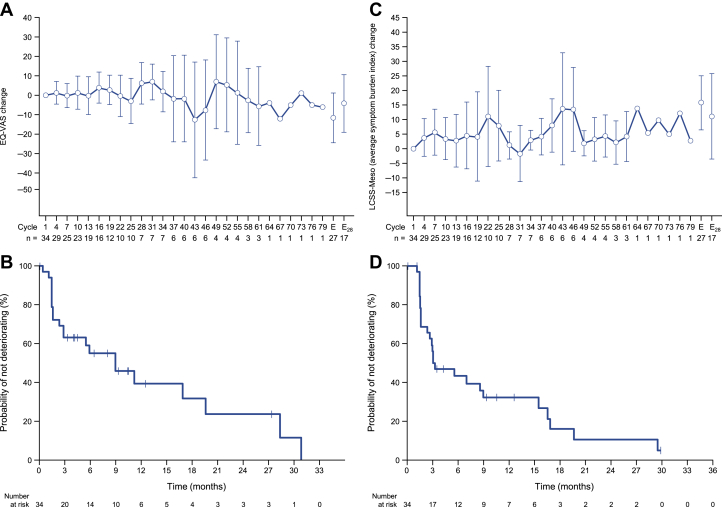
(*A, B*) Evolution of EQ-VAS and (*C, D*) LCSS-Meso average symptom burden index over time. Data are presented as means with 95% CIs. E, end of the treatment period (discontinuation); E_28_, 28 days after the end of the treatment period; EQ-VAS, EuroQOL visual analog scale; LCSS-Meso, Lung Cancer Symptom Scale for mesothelioma.

QOL was evaluated in terms of the EQ-VAS and LCSS-Meso symptom burden scale. Both outcomes were maintained over time among patients with available data (Fig. 4*A–D*).

### Safety

We previously reported that TRAEs occurred in 26 patients (76.5%), including grade 3 to 4 TRAEs in 11 (32.4%) by the cutoff date of March 14, 2018,^5^^,^^8^ and no additional TRAEs were observed thereafter until the cutoff date of November 12, 2019. There were no grade 5 TRAEs. The most common TRAEs were rash (six patients), lipase increased (five patients), and diarrhea and amylase increased (four patients each). Other TRAEs that occurred in at least two patients are listed in Table 2. Grade 3 or 4 TRAEs included lipase increased in four patients and diarrhea, amylase increased, and pneumonitis in two patients each.

**Table 2:** TRAEs in Two or More Patients (N = 34)

AE	Any Grade	Grades 3–4
Any	26 (76.5)	11 (32.4)
Most common AEs by preferred term (in ≥2 patients)		
Rash	6 (17.6)	1 (2.9)
Lipase increased	5 (14.7)	4 (11.8)
Diarrhea	4 (11.8)	2 (5.9)
Amylase increased	4 (11.8)	2 (5.9)
Stomatitis	3 (8.8)	1 (2.9)
Weight decreased	3 (8.8)	1 (2.9)
Decreased appetite	3 (8.8)	1 (2.9)
Fatigue	3 (8.8)	0 (0.0)
Malaise	3 (8.8)	0 (0.0)
Arthralgia	3 (8.8)	0 (0.0)
Pneumonitis	2 (5.9)	2 (5.9)
Interstitial lung disease	2 (5.9)	1 (2.9)
Hypothyroidism	2 (5.9)	0 (0.0)
Nausea	2 (5.9)	0 (0.0)
Vomiting	2 (5.9)	0 (0.0)
Mucosal inflammation	2 (5.9)	0 (0.0)
Pyrexia	2 (5.9)	0 (0.0)
Lymphocyte count decreased	2 (5.9)	0 (0.0)
Rash maculopapular	2 (5.9)	0 (0.0)

*Note:* Data are presented as n (%).

AE, adverse event; TRAE, treatment-related AE.

## Discussion

The MERIT study evaluated the efficacy and safety of nivolumab in Japanese patients with MPM, and led to the approval of nivolumab for this indication in Japan. Until now, long-term survival rates of patients with MPM have remained poor, with limited benefit of chemotherapy. For example, second-line pemetrexed in combination with best supportive care (8.4 versus 9.7 months for best supportive care alone)^9^ did not elicit marked improvements in OS. The introduction of ICIs has improved the prognosis of MPM. In the MAPS2 study, which enrolled patients with relapse after one or two lines of therapy, the median OS in nivolumab-treated patients was 11.9 months from the time of randomization (median follow-up of 20.1 months in the overall study population).^10^ Therefore, we analyzed the OS and PFS at a 3-year follow-up in the MERIT study. We observed a promising long-term survival of nivolumab-treated patients with a 3-year OS rate of 23.5%.

Although PD-L1 expression status was associated with the ORR, there were no significant differences in OS or PFS at 2 or 3 years between PD-L1–positive and PD-L1–negative patients. These results suggest that long-term survival in patients with nivolumab-treated MPM is not dependent on PD-L1 expression status. However, owing to the small number of patients, our findings may warrant confirmation in a future study with a larger number of patients or using a patient registry.

The histologic subtype of MPM is considered to be a prognostic factor for MPM, because patients with the biphasic or sarcomatoid histologic subtypes typically have worse prognosis after chemotherapy than patients with the epithelioid histologic subtype.^11^^,^^12^ In the present analyses, the survival outcomes, especially PFS, were quite favorable in the patients with nonepithelioid subtypes. Furthermore, as in our previous report,^5^ the ORR was also more favorable in patients with the nonepithelioid subtypes relative to that in patients with the epithelioid subtype. Thus, patients with nonepithelioid histologic subtypes tended to have better outcomes, although the reason for this is unknown. Further research is needed to investigate whether genomic alterations may explain the differences in survival with nivolumab between patients with nonepithelioid and epithelioid subtypes of MPM.

It is noteworthy that eight patients were alive at 3 years. There were no marked differences in patient characteristics between 3-year survivors and nonsurvivors except for ECOG PS at baseline.

Beyond assessing the efficacy of nivolumab in terms of tumor responses, we also examined its impact on QOL. We found that QOL, measured using the EQ-VAS and LCSS-Meso symptom burden index, was maintained over time in this cohort of nivolumab-treated patients. The stability of QOL in nivolumab-treated patients observed here may reflect the potential clinical benefit of nivolumab in terms of long-term survival, especially in responders.

The MERIT study also monitored the safety of nivolumab in patients with MPM. Of note, despite the longer follow-up of patients in the present analyses, we detected no additional TRAEs (any grade or grades 3–4) since the previous cutoff date,^5^^,^^8^ supporting the long-term safety of nivolumab in this patient population.

Another promising strategy for the treatment of MPM involves combining nivolumab with ipilimumab, a CTLA-4 antibody. This strategy was tested in the CheckMate 743 study, in which nivolumab plus ipilimumab significantly extended OS compared with chemotherapy (median: 18.1 versus 14.1 months, hazard ratio = 0.74, *p* = 0.002) with a median follow-up of 29.7 months.^13^ Thus, this combination is expected to become a standard of care for MPM in the future. However, nivolumab monotherapy after second-line treatment may be useful for ICI-naive patients.

Our findings should be discussed in the context of the limitations of the study, notably the single-arm design and the sample size (34 patients). Furthermore, the subgroups included in the analyses of overall response and survival were small, which might introduce some bias because the study was not powered to detect differences among subgroups. Therefore, we must take care when generalizing the results to a broader population of patients treated with nivolumab in clinical practice, and our findings should be confirmed in future studies with more patients.

In conclusion, the 3-year follow-up of the MERIT study reveals the longer-term efficacy and safety of nivolumab with survival for more than 3 years in some patients and a clinical benefit as second- or third-line therapy for patients with MPM.

## Data Availability

Qualified researchers may request Ono to disclose individual patient-level data from clinical studies through the following website: ClinicalStudyDataRequest.com. For more information on Ono’s Policy for the Disclosure of Clinical Study Data, please see the following website: https://www.ono.co.jp/eng/rd/policy.html.
