# Supplementary material for: Clinical Efficacy and Safety of Nivolumab in Japanese Patients With Malignant Pleural Mesothelioma: 3-Year Results of the MERIT Study
Source: JTO Clin Res Rep. 2020 Dec 29;2(3):100135. doi: 10.1016/j.jtocrr.2020.100135 (PMC8474205; doi:10.1016/j.jtocrr.2020.100135)
Supplement: Supplemental Materials [file mmc1.pdf]

Figure S1. Percent change in the sum of tumor sizes of the target lesion (central assessment, modified RECIST)

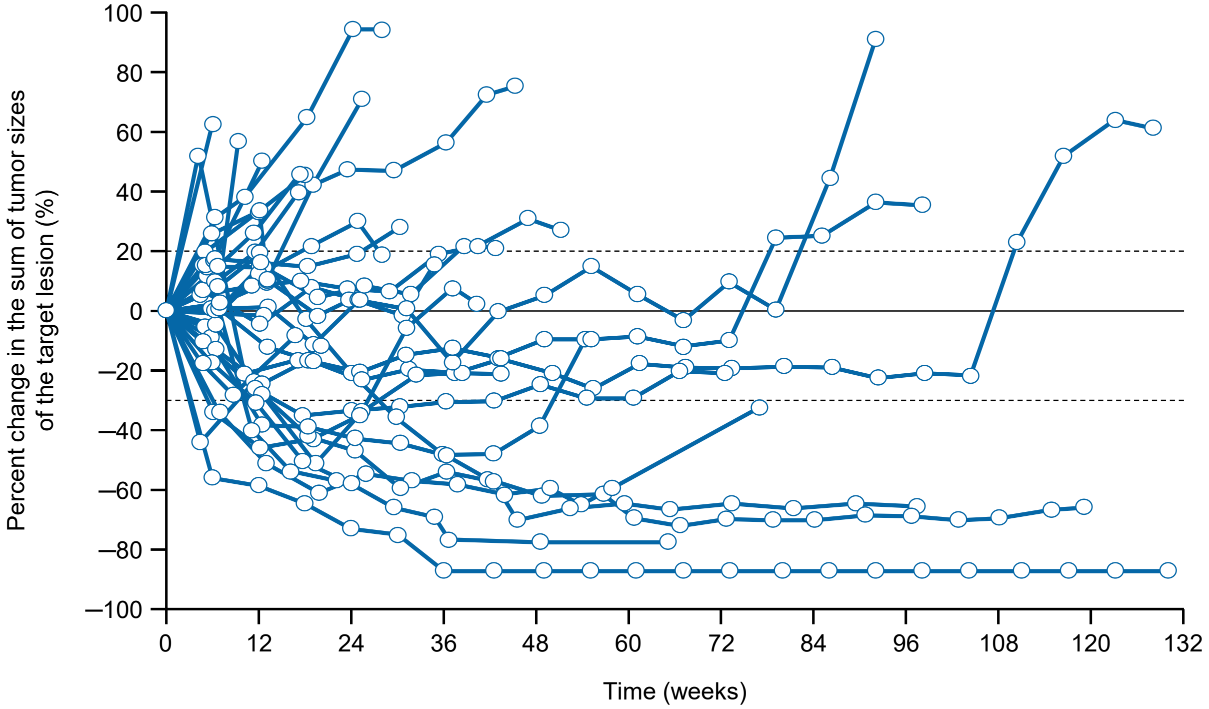

Figure S2. Landmark analysis of overall survival according to the best overall response at 3 months in patents who survived for ≥3 months. CI, confidence interval; HR, hazard ratio; OS, overall survival; PD, progressive disease; PR, partial response SD, stable disease

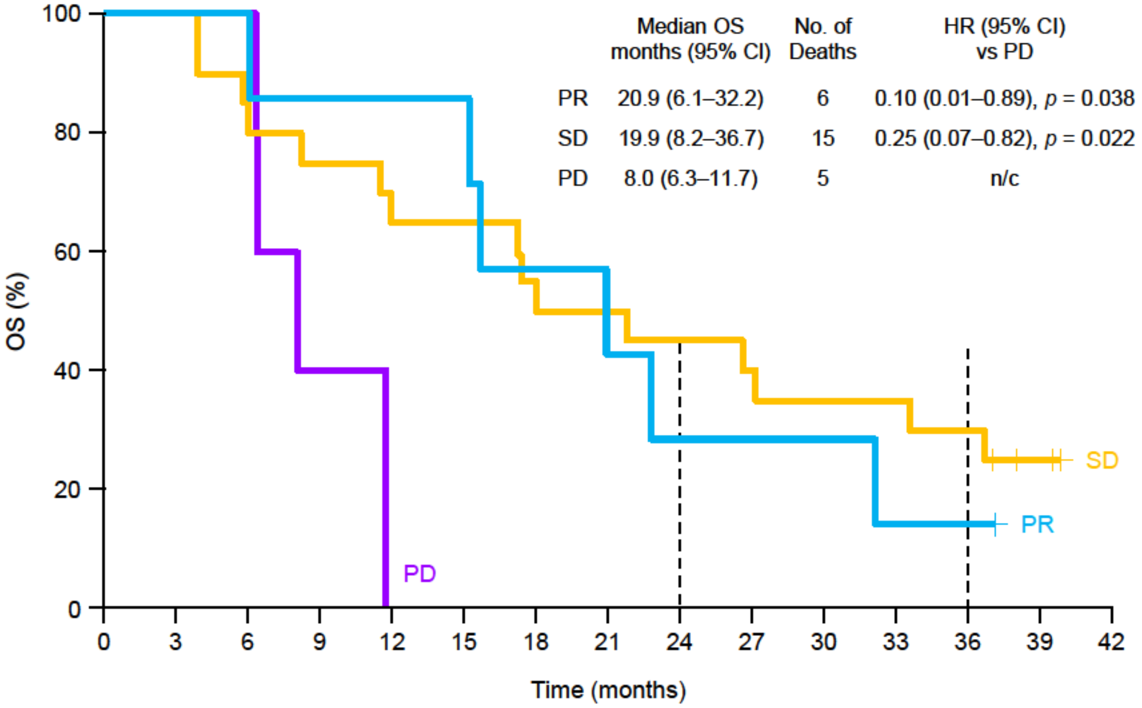

	0	3	6	9	12	15	18	21	24	27	30	33	36	39	42
PR	7	7	7	6	6	6	4	3	2	2	2	1	1	0	0
SD	20	20	16	15	13	13	11	10	9	8	7	7	6	2	0
PD	5	5	5	2	0	0	0	0	0	0	0	0	0	0	0

Table S1. Baseline demographic and clinical characteristics

	Nivolumab N = 34
Sex	
Male	29 (85)
Female	5 (15)
Age, years, median (range)	68.0 (43–78)
Body mass index, kg/m^2, median (range)	22.1 (15.8–29.0)
Number of prior treatment(s)	
1	24 (71)
2	10 (29)
Performance status	
0	13 (38)
1	21 (62)
Previous systemic therapy	
First line	
Pemetrexed + cisplatin or carboplatin	31 (91)
Pemetrexed + cisplatin + BBI608	2 (6)
Pemetrexed + cisplatin + bevacizumab	1 (3)
Second line	
Gemcitabine	3 (9)
Pemetrexed + cisplatin or carboplatin	3 (9)
Pemetrexed	2 (6)
Other	2 (6)
PD-L1 status	
≥1%	20 (59)
<1%	12 (35)
NA	2 (6)
Histological subtype	
Epithelioid	27 (79)
Biphasic	4 (12)
Sarcomatoid	3 (9)

Reprinted from Okada M, Kijima T, Aoe K, et al. Clinical efficacy and safety of nivolumab: results of a multicenter, open-label, single-arm, Japanese Phase II study in malignant pleural mesothelioma (MERIT). *Clin Cancer Res*. 2019;25:5485-5492

Data are n (%), unless otherwise stated

PD-L1, programmed death ligand-1; NA, not assessable

Table S2. Subsequent systemic therapies (N = 34)

	**n (%)**
**Any treatment**	**18 (52.9)**
Nivolumab	3 (8.8)
Pemetrexed	3 (8.8)
CBDCA + Pemetrexed	3 (8.8)
Investigational drug	3 (8.8)
Gemcitabine	2 (5.9)
Vinorelbine	1 (2.9)
CBDCA + Irinotecan	1 (2.9)
CDDP + Pemetrexed	1 (2.9)
Unknown*	1 (2.9)

*Patients whose subsequent treatment was unknown

CBDCA, carboplatin; CDDP, cisplatin

Table S3. Comparisons of 3-year survivors and non-survivors

	**3-year survivors (N = 8)**	**Non-3-year survivors (N = 26)**	**p-value**
Age	63.5 (43–75)	70.0 (54–78)	0.071[a]
Sex			0.309[b]
Male	8 (100.0%)	21 (80.8%)	
Female	0 (0.0%)	5 (19.2%)	
BMI, kg/m^2	22.27 (19.6–27.9)	21.86 (15.8–29.0)	0.591[a]
Number of prior treatment(s)			1.000[b]
1	6 (75.0%)	18 (69.2%)	
2	2 (25.0%)	8 (30.8%)	
ECOG PS			0.033[b]
0	6 (75.0%)	7 (26.9%)	
1	2 (25.0%)	19 (73.1%)	
PD-L1 status			0.379[b]
<1%	4 (50.0%)	8 (30.8%)	
≥1%	3 (37.5%)	17 (65.4%)	
Histologic subtype			0.315[b]
Epithelioid	5 (62.5%)	22 (84.6%)	
Biphasic or Sarcomatoid	3 (37.5%)	4 (15.4%)	
BOR			0.583[b]
PR	2 (25.0%)	8 (30.8%)	
Stable disease	4 (50.0%)	9 (34.6%)	
PD	1 (12.5%)	8 (30.8%)	
BOR			0.640[b]
PR + Stable disease	6 (75.0%)	17 (65.4%)	
PD	1 (12.5%)	8 (30.8%)	

Data are presented as median (range) or n (%)

[a]Wilcoxon rank sum test and [b]Fisher's exact test were used for comparisons between 3-year survivors and non-3-year survivors

BMI, body mass index; ECOG PS, Eastern Cooperative Oncology Group performance status; PD-L1, programmed death ligand-1; BOR, best overall response; PR, partial response; PD, progressive disease
